# Stress Responses Among Individuals with Spiritual Struggles in Hungary: An Experimental Study

**DOI:** 10.1007/s10943-023-01819-2

**Published:** 2023-04-25

**Authors:** Szabolcs Kéri

**Affiliations:** 1 Department of Cognitive Science, Budapest University of Technology and Economics, Egry J. Str. 1, Budapest, 1111 Hungary; 2 National Institute of Mental Health, Neurology and Neurosurgery - Nyírő Gyula Hospital, Budapest, Hungary; 3 Faculty of Medicine, Department of Physiology, University of Szeged, Szeged, Hungary

**Keywords:** Religious struggle, Spirituality, Stress, Trier social stress test, Cortisol, Lateralized frontal activity

## Abstract

Individuals with a Religious or Spiritual Problem (RSP), as defined in the DSM-5, experience distress associated with faith-related moral dilemmas, existential meaning, and transpersonal attitudes toward other people. It is unclear whether a RSP reflects a generally heightened stress reactivity or whether the stress response is confined to religious and spiritual contexts. To elucidate this issue, we measured behavioral and physiological responses during social-evaluative stress (public speaking—Trier Social Stress Test) and in religious/spiritual contexts (Bible reading and listening to sacred music) in 35 individuals with RSP and 35 matched participants. We found no stress reduction in the religious/spiritual context in RSP, as indicated by increased heart rate, saliva cortisol, and relatively higher left than right frontal activity. Religious stimuli evoked physiological stress responses in RSP. Contrary to the physiological parameters, participants with RSP reported less anxiety in the religious/spiritual context. Religious individuals with and without RSP showed similar stress responses during public speaking. Religious individuals without RSP displayed reduced stress responses in the religious/spiritual context. These results indicate that specific physiological distress in religious/spiritual contexts should be considered in the psychological care of RSP.

## Introduction

A wealth of evidence indicates that religion and spirituality positively affect physical and mental health (Koenig et al., 2012). However, religious and spiritual struggle negatively impacts everyday life. Effective coping with religious/spiritual crises carries a potential for transformation and growth (Exline & Rose, 2005; Grof & Grof, 1990; Pargament et al., 2004). Religious and spiritual struggle results in anxiety, uncertainty, anguish, despair, and social isolation, substantially influencing beliefs, attitudes, values, and identity (Greenfield & Marks, 2007; Hall, 1997). Adverse life events and transformation experiences raise fundamental issues regarding one's relationship with the divine in the form of questioning faith, moral dilemmas, existential significance, ultimate meaning, and attitudes toward other humans (Pomerleau et al., 2020).

The publication of the fourth edition of the Diagnostic and Statistical Manual of Mental Disorders (DSM-IV, 1994) constituted a landmark in the clinical interpretation of Religious or Spiritual Problem (RSP), clearly distinguishing it from psychopathological phenomena (Lukoff, 1998; Prusak, 2016). A section in the DSM-5 focusing on problems related to psychosocial, personal, and environmental circumstances defines RSP: “This category is relevant when the focus of clinical attention is a religious or spiritual problem. Examples include distressing experiences that involve loss or questioning of faith, problems associated with conversion to a new faith, or questioning of spiritual values that may not necessarily be related to an organized church or religious institution.” (American Psychiatric Association, 2013). The category of RSP is also included in the revised version of DSM-5 (DSM-5-TR) (American Psychiatric Association, 2022).

The concept of RSP is similar to religious/spiritual struggle, defined as "tensions, strains, and conflicts about what people hold sacred" (Exline & Rose, 2005; Exline et al., 2014; Pargament & Exline, 2022). Comparing DSM-5 RSP and religious/spiritual struggle is of particular importance. For example, the “loss or questioning of faith” and “conversion to new faith” in DSM-5 RSP correspond to divine struggles (“Anger or disappointment with God, and feeling punished, abandoned, or unloved by God.”) and doubt-related struggles (“Feeling confused about religious/spiritual beliefs, and feeling troubled by doubts or questions about religious/spiritual.”) (Exline et al., 2014). Moreover, the “questioning of spiritual values” in the DSM-5 is similar to moral struggles (“Tensions and guilt about not living up to one’s higher standards and wrestling with attempts to follow moral principles.”) and struggles of ultimate meaning (“Concerns that life may not really matter, and questions about whether one’s own life has deeper meaning.”) (Exline et al., 2014). However, evidence suggests a high correlation among different dimensions of religious/spiritual struggle (divine, demonic, doubt-related, moral, ultimate meaning, and interpersonal), revealing a general religious/spiritual struggle factor (Stauner et al., 2016).

Most mental health professionals agree that RSP does not necessarily indicate a mental disorder, but religious and spiritual struggle hinders mental health (Pargament & Exline, 2021). However, it is unknown whether RSP, as defined in the DSM-5, is specific to faith-related problems or part of a generally heightened stress responsiveness. In other words, the question may arise as to whether individuals with RSP demonstrate circumscribed stress reactivity in religious contexts or are more susceptible to stressful situations.

Stress reactivity can be investigated at multiple levels, including subjective experiences, autonomic nervous system (e.g., heart rate acceleration due to increased sympathetic nervous system activity), endocrine changes (heightened cortisol secretion in the adrenal cortex), and frontal brain activation. Critically, stress-related activation of the hypothalamic–pituitary–adrenal axis results in increased cortisol secretion, which affects metabolism, inflammation, immune responses, cardiovascular functions, and homeostatic balance (Sapolsky, 2021).

Recently, it has been proven that a multidisciplinary approach is highly feasible in elucidating the link between religious worldviews and health. For example, Schnell et al. (2020) used the Trier Social Stress Test (TSST) to explore the relationship between worldview security and social stress responsiveness. The TSST is a widespread experimental paradigm in psychological sciences to assess the reactivity of the sympathetic nervous system and the hypothalamic–pituitary–adrenal stress axis (Bali & Jaggi, 2015; Dickerson & Kemeny, 2004; Narvaez Linares et al., 2020). During the stress induction phase of the TSST, participants perform a mental arithmetic task before a jury, similar to a typical examination or public speaking. In addition to the subjective experiences of atheists, religious individuals, and spiritual seekers during the TSST, Schnell et al. (2020) also measured cardiovascular reactivity (blood pressure and heart rate) and endocrine responses (saliva cortisol). The key finding was that existential search and worldview instability positively correlated with systolic blood pressure, increased heart rate, and saliva cortisol, which are putative markers of risk for cardiovascular and metabolic diseases and mood and anxiety disorders (Schnell et al., 2020).

However, studies focusing on RSP have not investigated physiological changes and stress-related brain activity. The alpha-rhythm asymmetry in left vs. right frontal areas in the electroencephalogram (EEG) is a well-known measure of cortical activity related to emotional and cognitive processing during stress. Several studies revealed that individuals with greater right than left frontal resting-state neuronal activity experience higher negative feelings and emotions (Allen & Cohen, 2010; Coan & Allen, 2004; Reznik & Allen, 2018; Tops et al., 2017). Moreover, higher right frontal activity predicts the intensity of the physiological stress response (increased heart rate and cortisol secretion) (Ma et al., 2021; Zhang et al., 2018). Therefore, higher baseline left than right frontal activity is a marker of more efficient coping with adverse events, resulting in better psychological well-being (Urry et al., 2004). However, pronounced left frontal brain activity may also indicate an overload of cognitive coping mechanisms (Davidson, 2004). When individuals faced a social-evaluative threat and uncontrollability in a public speaking test, higher left frontal activity marked the intensity of endocrine stress responses (increased cortisol secretion) (Düsing et al., 2016). At the level of subjective experiences and cognitive processes, enhanced left frontal activity indicates action orientation (to approach goals in appetitive or aversive situations), heightened hesitation and decisional uncertainty, repetitive thought patterns, and rumination to cope with the stressful situation effortfully (Düsing et al., 2016; Haehl et al., 2021; Roth & Cohen, 1986).

An essential and unexplored question is how individuals with RSP react in stressful situations. To evaluate the specificity of stress reactivity, we compared two conditions: exposition to challenging everyday situations (public speaking) and participating in religious/spiritual activities. For example, individuals with RSP may feel overwhelmed when reading Bible verses relative to people with stable religiosity who experience calming and supporting Bible reading. On the other hand, individuals with RSP may feel the same stress level as religious people without RSP during mundane situations (e.g., social evaluation in public speaking).

Therefore, the hypotheses of the present study were the following:

### Hypothesis 1

Individuals with and without RSP show similar anxiety, heart rate, cortisol secretion, and lateralized frontal brain activity during the TSST.

### Hypothesis 2

Individuals with RSP display increased anxiety, accelerated heart rate, enhanced cortisol secretion, and higher left frontal activity than control participants without RSP in a religious context (Bible reading and listening to sacred music).

## Material and Method

### Participants, Interviews, and Rating Scales

We enrolled 35 individuals with RSP and 35 matched participants who did not experience RSP from Hungary’s Roman Catholic, Protestant, and Pentecostal communities. Participants with RSP attended the pastoral psychological care service at the Nyírő Gyula Hospital (Budapest, Hungary). All of them defined themselves as highly religious believers. People with RSP sought help because of questioning their faith following adverse life events. They reported moral dilemmas concerning work and political commitment, conflicts related to differences in religious views, and feeling guilty because of prohibited sexual behavior. RSP resulted in existential anxiety and interpersonal conflicts.

To evaluate possible psychiatric disorders and to define RSP, we used a structured clinical interview for DSM-5 (Diagnosis and Statistical Manual of Mental Disorders—5) (First et al., 2016). The cultural impact on clinical presentation is especially relevant in RSP. Therefore, we administered each participant the DSM-5 Cultural Formulation Interview (CFI) (American Psychiatric Association, 2013). We did not include individuals with psychiatric disorders in the present study. However, all participants meet the DSM-5 definition of "Problems related to other psychosocial, personal, and environmental circumstances" (Religious or Spiritual Problem, [RSP, code: V62.89] (American Psychiatric Association, 2013).

We also assessed general cognitive functioning [Wechsler Adult Intelligence Scale-IV, WAIS-IV (Wechsler, 2008)], socioeconomic status [Hollingshead Four-Factor Index of Socioeconomic Status, SES (Hollingshead, 1975)], subjective depressive experiences [Beck Depression Inventory-II, BDI-II (Beck et al., 1996)], and anxiety [Beck Anxiety Inventory, BAI (Beck et al., 1988)] (Perczel-Forintos et al., 2018; Rózsa et al., 2010).

To delineate the religious behavior of the participants, we administered the modified Duke University Religiosity Index (DUREL), which assesses organized religious activity, individual religious activity, and intrinsic religiosity (Koenig & Büssing, 2010). During the recruitment of individuals with and without RSP, we systematically screened for potential confounding factors in stress measurements, including nicotine, caffeine, and alcohol intake, contraception use, body mass index, chronic diseases (e.g., cardiovascular and metabolic diseases), and working night shifts (Narvaez Linares et al., 2020). Table 1 depicts the characteristics of the participants.

**Table 1:** Characteristics of the participants

	Religious or Spiritual Problem (*n* = 35)	Control participants (*n* = 35)	*t*	*p*
Gender (male/female)	19/16	17/18	–	–
Number of Smokers	10	9	–	–
Number of chronic disease	8	7	–	–
Use of contraceptives	8	9	–	–
Age (years)	34.2 (12.3)	37.3 (10.0)	− 1.14	0.26
Education (years)	12.9 (4.4)	13.2 (4.3)	− 0.25	0.81
Wechsler Adult Intelligence Scale—IV (WAIS-IV)	104.1 (14.9)	106.2 (11.1)	− 0.67	0.50
Hollingshead Four-Factor Index (socioeconomic status)	31.0 (14.8)	32.9 (12.3)	− 0.58	0.56
*Duke University Religiosity Index (DUREL)*				
Organized Religious Activity (1–5 points)	3.3 (1.5)	3.1 (1.4)	0.42	0.68
Non-organized (private) religious activity (1–5 points)	3.5 (1.5)	3.4 (1.5)	0.16	0.88
Intrinsic religiosity (1–5 points)	3.9 (1.1)	3.7 (1.4)	0.84	0.40
Beck Depression Inventory (BDI-II)	15.7 (7.2)	6.8 (4.9)	6.10	< 0.001
Beck Anxiety Inventory (BAI)	9.9 (7.9)	6.4 (4.6)	2.22	< 0.05

Data are mean (standard deviation) except for gender distribution. The two groups were compared with two-tailed *t*-tests

### Outline of the Procedure

#### Main Assessment

Participants received the interviews, rating scales, and a detailed procedure description in the first session. Upon agreement, we arranged a second session a few days later when we conducted the stress measurements. The sessions started between 11 and 13 h and consisted of three phases: baseline, stress induction, and recovery. During the baseline phase (30 min), we presented the essential information and provided sufficient time to acclimatize. Next, at the end of the baseline phase, we conducted the measurements (t_[baseline]_): visual analog scale (VAS) for stress and anxiety, saliva cortisol, heart rate, and EEG. Immediately after this, volunteers participated in the stress induction phase (20-min), which included the TSST (simulated job interview and arithmetic calculations). At the end of the stress induction phase, we repeated the measurements used at the end of the baseline phase (t_[stress]_). Finally, we administered a recovery phase (40 min) when participants relaxed and silently read verses from the Bible and listened to relaxing religious piano music. Again, all measurements were repeated at the end of the recovery phase (t_[recovery]_).

#### Replication

We asked the participants to visit the laboratory approximately two weeks after the main assessment. In this case, we performed the baseline and the recovery phase of the original assessment (religious context) without social-evaluative stress. The main question of the replication experiment was whether the religious context (Bible reading and religious music) alone could elicit a stress response in RSP. Thirty-one individuals with RSP and 32 matched participants without RSP were willing to complete the replication phase from the original sample included in the main assessment.

### Stress Induction and Recovery

We used a public-speaking procedure based on the TSST, which has been shown to evoke social-evaluative stress with increased cortisol secretion and EEG signatures (Dickerson & Kemeny, 2004; Düsing et al., 2016). We considered the newest available protocol for TSST, including a standardized background questionnaire for confounding variables and physiological recordings (Narvaez Linares et al., 2020).

Participants were informed that the test measures their public speaking and cognitive abilities in front of an expert committee. They performed a simulated job interview and then arithmetic calculations (subtracting 13 from a random number > 4800) during the procedure. In the original version, volunteers spoke in front of an audience, but in our study, participants performed in front of a video camera (Düsing et al., 2016). The public-speaking phase was followed by a recovery period (40-min), during which participants listened to the Hillsong worship piano (Musselman, 2019) and silently read calming verses from the Bible (Zondervan, 2019). The subjective experience of stress and anxiety was measured using a VAS (visual analog scale comprising a horizontal line between 0 and 10 points on the computer screen; 0—no stress and anxiety, 10—the highest stress level and anxiety). We administered the VAS before the stress induction phase (t_[baseline]_), immediately after the stress induction phase (t_[stress]_), and at the end of the recovery period (t_[recovery]_).

### EEG Measurements

We recorded resting-state EEG at three time points (t_[baseline]_, t_[stress]_, t_[recovery]_). Each measurement included eight 1-min resting periods (four occasions with eyes open and four with eyes closed, counterbalanced across subjects). EEG was recorded and processed with a NEUVO—CURRY 8X-system with a 256-channel Quik-Cap Neo Net (high-density EEG cap, Ag/AgCl electrodes, four bipolar leads for vertical and horizontal electrooculogram, extended international 10–20 system) (Compumedics, NeuroScan). The electrode impedances were checked (< 5 kΩ, homologous bilateral leads: < 1 kΩ). The sampling rate was 500 Hz. For data processing, we used the EEGLAB interactive MATLAB toolbox (Schwartz Center for Computational Neuroscience, University of California). Following built-in automatic and manual artifact reduction, the 1-min epochs were segmented in 2-s periods with 75% overlap between epochs (epoch amplitudes <  ± 75 μV) and were low-pass filtered at 30 Hz (Düsing et al., 2016). We used Fourier transformation to generate the spectral power (μV^2^) (resolution of 0.488 Hz) in the alpha band (8–13 Hz). Every 1 min EEG registration included at least 20 2-s epochs, and power density was averaged using all epochs. We used logarithmic transformation (ln) for averaged power density values. We calculated the frontal asymmetry index for the 1-min epochs by subtracting the logarithmically transformed alpha frequency of left electrode sites from homologous right leads (e.g., F4-F3, F8-F7). Higher alpha-asymmetry scores indicate relatively more robust left-sided frontal activation (Duan et al., 2019; Düsing et al., 2016).

### Physiological Measurements (Heart Rate and Saliva Cortisol)

Heart rate was measured with Frontier X chest-work heart monitor (Fourth Frontier), which records a high-quality, continuous electrocardiogram (ECG) validated against a GE Holter Monitor. We registered heart rates at three time points (t_[baseline]_, t_[stress]_, t_[recovery]_). All data were processed offline. Two measurements were conducted at each time point, and the average was analyzed.

Saliva samples were also collected from each participant at the same three time points (t_[baseline]_, t_[stress]_, t_[recovery]_). We used SalivaBio Passive Drool Method for saliva sample collection and stored the samples at − 10 °C. Free salivary cortisol concentrations were measured by using Salimetrics immunoassays (assay range: 0.012–3.00 µg/dL; sensitivity: < 0.007 µg/dL) (Szőllősi, Pajkossy, Demeter, Kéri, & Racsmány, 2018). We analyzed two samples at each time point with excellent consistency (< 2% differences between the two samples). The average of the two samples was used for data analysis.

### Data Analysis

We used STATISTICA 13.5 (Tibco) for data analysis. Before considering parametric tests, all data were checked for normal distribution (Kolmogorov–Smirnov test) and homogeneity of variance (Levene’s test). Repeated-measures analyses of variances (ANOVAs) were conducted to determine the differences between individuals with and without RSP in anxiety-VAS, heart rate, saliva cortisol, and lateralized EEG activity at t_[baseline]_, t_[stress]_, and t_[recovery]_. The two groups were matched for confounding factors (e.g., smoking, exercising before participation, hours of sleep in the previous night, being a postmenopausal, acute or chronic illness, hormonal contraception) (Narvaez Linares et al., 2020; Schnell et al., 2020). Therefore, these factors were not included as covariates in the ANOVAs. Individuals with and without RSP differed in BDI-II and BAI scores; these measures were covariates in the ANOVAs. Two-tailed t-tests were performed to compare the demographic parameters and test scores. Tukey's HSD (honestly significant differences) tests were applied for post hoc analysis. The level of statistical significance was set at alpha < 0.05. Effect sizes *(ƞ*^*2*^*)* were also calculated for ANOVA main effects and interactions, and 95% confidence intervals were reported.

## Results

### Anxiety-VAS

#### Main Assessment

**Fig. 1:**
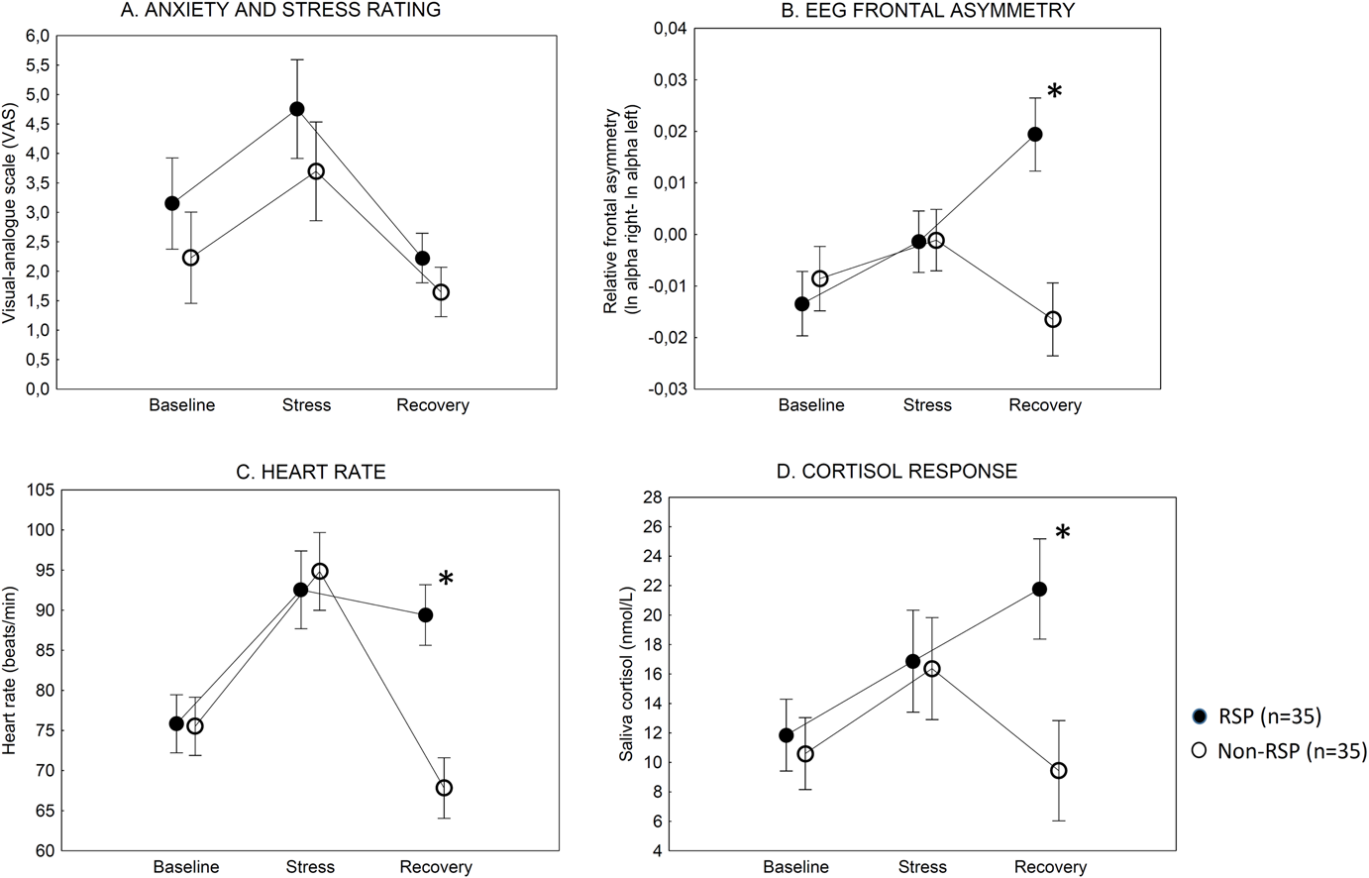
Behavioral and physiological measures in individuals with Religious or Spiritual Problem (RSP) and matched religious individuals without RSP (non-RSP). The stress phase included a public speech (social-evaluative stress). During the recovery phase, participants read calming Bible verses and listened to sacred music. Data are mean, and error bars indicate 95% confidence intervals. * *p* < .001, RSP vs. non-RSP, Tukey’s HSD tests

The ANOVA performed on the anxiety-VAS scores yielded significant main effects of group (RSP vs. non-RSP) (*F*(1,68) = 8.71, *p* < 0.01, *ƞ2* = 0.11) and test phase (t_[baseline]_, t_[stress]_, and t_[recovery]_) (*F*(2,136) = 95.22, *p* < 0.001; *ƞ2* = 0.25). The two-way interaction between the group and test phase was not significant (*p* = 0.50). However, Tukey's HSD tests indicated no significant differences between individuals with and without RSP at t_[baseline]_, t_[stress]_, and t_[recovery]_) (*p*s > 0.2). Both groups scored higher in the stress phase than in the baseline phase (t_[stress]_ > t_[baseline]_), and the anxiety-VAS scores declined in the recovery phase (t_[recovery]_ < t_[stress]_) (*p*s < 0.05) (Fig. 1A).

#### Replication

**Fig. 2:**
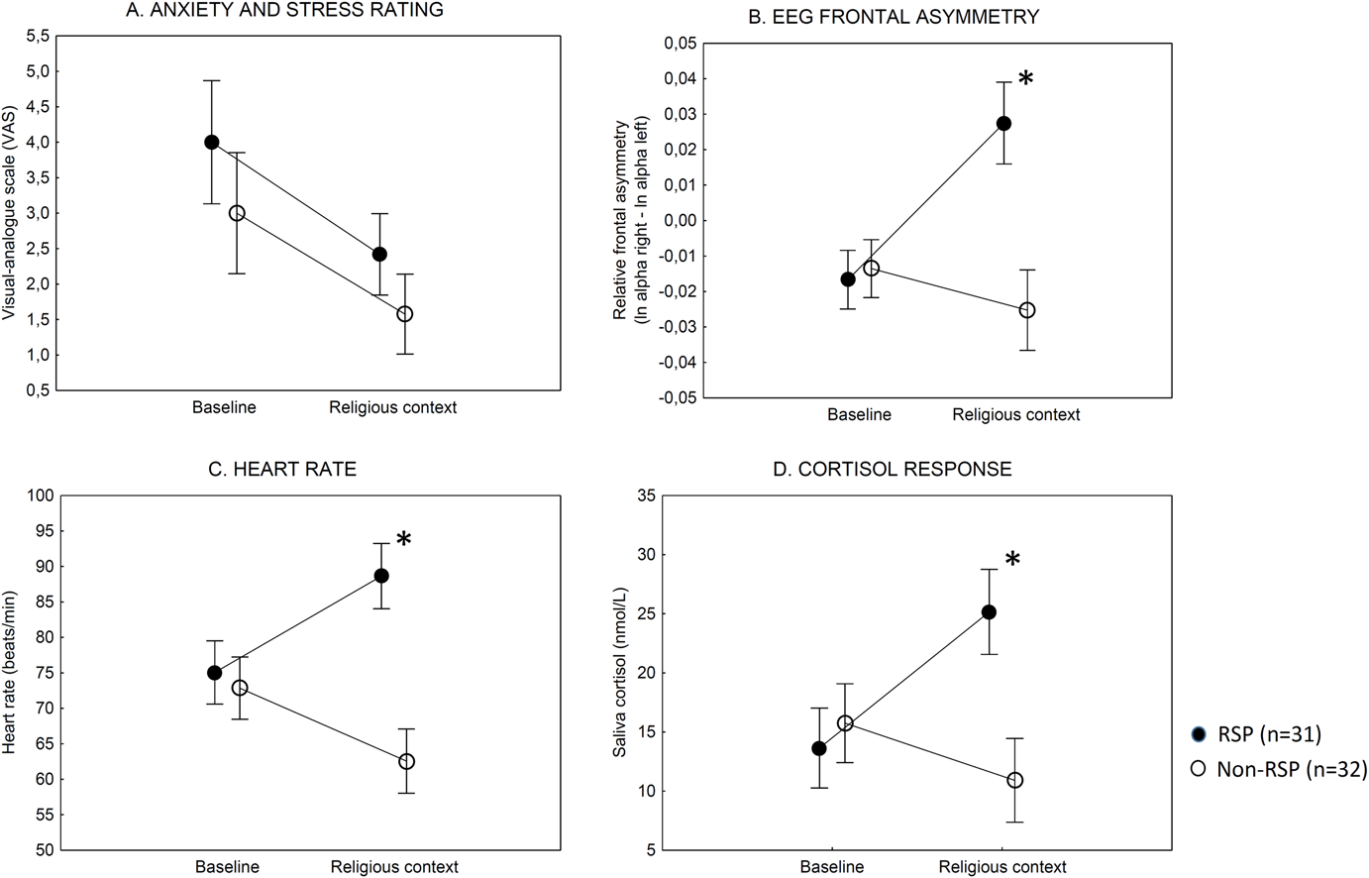
Behavioral and physiological measures from the replication assessment in individuals with Religious or Spiritual Problem (RSP) and matched religious individuals without RSP (non-RSP). The religious context included reading calming Bible verses and listening to sacred music. Data are mean, and error bars indicate 95% confidence intervals. *p < .001, RSP vs. non-RSP, Tukey’s HSD tests

There were significant main effects of group (*F*(1,61) = 6.30, *p* < 0.05, *ƞ2* = 0.09) (RSP vs. non-RSP) and test phase (baseline, religious context) (*F*(1,61) = 17.16, *p* < 0.001; *ƞ2* = 0.22). The two-way interaction between the group and test phase was not significant (*p* = 0.80). Tukey’s HSD tests revealed no significant differences between RSP and non-RSP participants. In both groups, we observed a reduction of anxiety-VAS scores during the religious context phase (*p*s < 0.05) (Fig. 2A).

### EEG Measures

#### Main Assessment

The ANOVA conducted on the frontal asymmetry index indicated significant main effects of group (*F*(1,68) = 14.92, *p* < 0.001, *ƞ2* = 0.18) and test phase (*F*(2,136) = 8.24, *p* < 0.001, *ƞ2* = 0.11). The two-way interaction was also significant (*F*(2,136) = 23.91, *p* < 0.001, *ƞ2* = 0.26). There were no significant differences between the RSP and non-RSP groups in the baseline and stress phases (t_[baseline]_ and t_[stress]_, *p*s > 0.05). However, individuals with RSP displayed significantly higher frontal asymmetry in the recovery phase relative to the non-RSP participants (t_[recovery]_, *p* < 0.001). It is noteworthy that in the RSP group, the frontal asymmetry values significantly increased in the recovery phase relative to the stress phase (t_[recovery]_ > t_[stress];_ *p* < 0.001). In the non-RSP group, we found the opposite effect: the frontal asymmetry index was lower in the recovery phase than in the stress phase (t_[recovery]_ < t_[stress]_, *p* < 0.05) (Fig. 1B).

#### Replication

We again found significant main effects of group (*F*(1,61) = 26.15, *p* < 0.001, *ƞ2* = 0.30) and test phase (baseline vs. religious context) (*F*(1,61) = 10.10, *p* < 0.001, *ƞ2* = 0.14). The two-way interaction was also significant (*F*(1,61) = 29.83, *p* < 0.001, *ƞ2* = 0.33). At baseline, there were no significant differences between RSP and non-RSP participants (*p* = 0.9), but in the religious context, individuals with RSP display markedly higher frontal asymmetry index (*p* < 0.001) (Fig. 2B).

### Physiological Measures: Heart Rate and Saliva Cortisol

#### Main Assessment

The ANOVA investigating heart rate indicated significant main effects of group (*F*(1,68) = 14.12, *p* < 0.001, *ƞ2* = 0.17) and test phase (*F*(2,136) = 45.21, *p* < 0.001, *ƞ2* = 0.40). The two-way interaction was significant (*F*(2,136) = 20.78, *p* < 0.001, *ƞ2* = 0.23). We found no significant differences between the RSP and non-RSP groups in the baseline and stress phases (t_[baseline]_ and t_[stress]_, *p*s > 0.05). However, individuals with RSP displayed significantly higher heart rate in the recovery phase relative to the non-RSP participants (t_[recovery]_, *p* < 0.001). In the RSP group, heart rate did not change in the recovery phase compared to the stress phase (t_[stress] =_ t_[recovery]_, *p* > 0.5). In the non-RSP group, heart rate returned to the baseline level in the recovery phase (t_[recovery] =_ t_[baseline]_ < t_[stress,]_, *p* < 0.001) (Fig. 1C).

The ANOVA performed on saliva cortisol indicated significant main effects of group (*F*(1,68) = 12.23, *p* < 0.001, *ƞ2* = 0.15), test phase (*F*(2,136) = 6.98, *p* < 0.01, *ƞ2* = 0.09), and an interaction between group and test phase (*F*(2,136) = 9.32, *p* < 0.001, *ƞ2* = 0.12). Similar to the heart rate, individuals with and without RSP differed only in the recovery phase when RSP individuals showed elevated saliva cortisol levels (*p* < 0.001). Significant decreases in saliva cortisol levels were only seen in participants without RSP (t_[recovery]_ < t_[stress]_, *p* < 0.05). In the RSP group, we observed paradoxically higher saliva cortisol concentrations in the recovery phase compared to the stress phase (t_[recovery]_ > t_[stress]_, *p* < 0.05) (Fig. 1D).

#### Replication

In the case of heart rate, there was a significant main effect of group (RSP vs. non-RSP) (*F*(1,61) = 38.50, *p* < 0.001, *ƞ2* = 0.39), and a significant interaction between group and task phase (baseline vs. religious context) (*F*(1,62) = 28.95, *p* < 0.001, *ƞ2* = 0.32). At baseline, we measured similar heart rate in RSP and non-RSP participants (*p* = 0.90). In the religious context, individuals with RSP displayed increased heart rate. In contrast, participants without RSP showed decreased heart rate (*p*s < 0.05) (Fig. 2C).

In the case of saliva cortisol, we found significant main effects of group (RSP vs. non-RSP) (*F*(1,61) = 9.29, *p* < 0.01, *ƞ2* = 0.13), task phase (baseline vs. religious context) (*F*(1,61) = 5.45, *p* < 0.05, *ƞ2* = 0.08), and a two-way interaction between them (*F*(1,61) = 32.94, *p* < 0.001, *ƞ2* = 0.35). The post hoc tests indicated that individuals with RSP showed increased cortisol secretion in the religious context (*p* < 0.001). In contrast, non-RSP participants did not display a similar change in cortisol secretion (*p* = 0.08, a tendency of decreased cortisol secretion in the religious context) (Fig. 2D).

## Discussion

The core finding of the present study was that individuals with RSP exhibited increased stress responses only in a religious context relative to matched religious people without RSP. We also found that during social-evaluative stress, the RSP and non-RSP groups showed similar responses on subjective anxiety ratings, physiological measures (cardiovascular activity and cortisol secretion), and lateralized frontal EEG activity. Moreover, when the social-evaluative stress situation was followed by a recovery phase in a religious context (Bible reading and sacred music), only people without RSP displayed alleviated stress responses. It is essential to underline that the results were replicated when the task was solely Bible reading and listening to sacred music without preceding social-evaluative stress, confirming that religious materials alone can be stressful at the physiological level in people with RSP.

The results from the present study are in accordance with the findings of Stauner et al. (2016). In this study, the authors identified a general factor in addition to the five components of religious/spiritual struggle (divine, demonic, interpersonal, moral, and doubt-related). The general factor showed a definitive correlation with religiousness but did not alter the correlation of the five factors with neuroticism, depression, anxiety, and stress (Stauner et al., 2016). The findings of Stauner et al. (2016) confirmed that religious/spiritual struggle is a psychological construct different from religiosity and stress.

Multiple factors contribute to the emergence of religious/spiritual struggle, including the negative appraisal of stressful situations, negative affectivity, and insecure and ambivalent attachment to God (Ano & Pargament, 2013). When adverse life events are appraised as a sacred loss, individuals experience intrusive thoughts, depression, and pronounced posttraumatic growth (appreciation of life, deepened relationships with others, spiritual change, new possibilities, and empowerment) (Pargament et al., 2005). Accordingly, RSP can be interpreted as a consequence of negative religious coping with stress and not general dispositional factors. It is important that RSP was linked to stress responses exclusively in a religious context. Individuals with RSP did not show unusually high responses in a mundane context (social stress).

Notably, there was an intriguing dissociation between subjective anxiety and physiological parameters in the RSP group. Although people with RSP reported a resolution of anxiety during Bible reading, their physiological responses and lateralized frontal EEG activity still indicated heightened stress levels. Decreased reported anxiety might be a form of social desirability because religiously committed people are explicitly or implicitly expected to experience positive emotions in a religious context. Indeed, it has been shown that social desirability biases personal reports on religious orientation, coping, and spiritual experiences (Jones & Elliott, 2017).

Positive religious coping helps deal with adverse life events, trauma, and loss by focusing on a sacred higher power, positive reframing, transcendent meaning, support, empowerment, and spiritual growth. In contrast, negative religious coping and struggle, closely related to DSM-5 RSP, are associated with adverse feelings, inner tension, anxiety, and strain (e.g., scrupulousness, punishment from the sacred higher power, awe, desolation, and spiritual discontent) (Exline & Rose, 2005; Pargament, 2001; Pargament et al., 1998, 2011).

Not surprisingly, positive religious coping predicts beneficial mental and physical health outcomes, whereas prolonged religious struggle is related to poor health and worse well-being (Magyar-Russell et al., 2014; Pargament et al., 2001; Ramirez et al., 2012). Abnormal cortisol secretion, circulating pro-inflammatory cytokines, and low-grade peripheral inflammation are critical biological factors linking religious struggle (negative affectivity) and unfavorable health outcomes because these factors are implicated in cardiovascular and metabolic diseases, immune dysfunctions, and mental disorders (Ai et al., 2010a, 2010b; Ai et al., 2010a, 2010b; Exline & Rose, 2005; Ironson et al., 2002; Sapolsky, 2021; Sephton et al., 2001). For example, Tobin and Slatcher (2016) obtained data on religious participation, religious coping, and diurnal cortisol levels from 1470 subjects from the Midlife in the United States (MIDUS) study. Findings indicated that religious struggle mediated the positive association between religious participation and healthier diurnal cortisol secretion. In other words, intensive religious attendance predicted low religious struggle a decade later, associated with a regular pattern of daily cortisol secretion (Tobin & Slatcher, 2016). Our present interventional study adds novel data to these large-scale observational studies, indicating that RSP is not a condition with a generally elevated stress response. Individuals with RSP exhibit the same stress response as non-RSP people in a social-evaluative situation, but religious practice and experience have no stress-reducing effect. Instead, we observed enhanced physiological stress responses in a religious context in RSP.

As discussed above, religious struggle in RSP and elevated cortisol levels may impact mental health and physical well-being. At the level of cognitive processing, perseverative thinking and rumination on negative feelings are typical features of religious struggle (Pargament, 2001). Lateralized frontal activity is a physiological marker of coping and self-control attempts, including approach motivation, perseverative cognition, and affect regulation. These critical cognitive factors in RSP are related to cortisol secretion, health, and well-being (Davidson, 2004; Düsing et al., 2016; Pitchford & Arnell, 2019; Urry et al., 2004).

In accordance with previous findings, we found increased left relative to right frontal activity during stress, which may reflect the apprehension of negative feelings and preoccupation with future outcomes (Carter et al., 1986; Düsing et al., 2016; Engels et al., 2007). A critical finding was that in individuals with RSP, increased left frontal activity did not return to the baseline level in the religious recovery phase. Paradoxically, we observed that left frontal activity further increased in the religious recovery phase compared to social-evaluative stress in RSP, which indicates an additional cognitive load during Bible reading. Religious individuals without RSP displayed the opposite effect (reduced left frontal activity in the recovery phase), suggesting that Bible reading and sacred music attenuated stress-related cognitive efforts in their case.

These experimental findings may be relevant in understating the primary mechanisms of religious coping, which refers to how individuals use their religious beliefs, practices, and resources to manage the challenges and stresses of life (Pargament, 2001). It involves turning to religious or spiritual beliefs, rituals, and practices as a source of comfort, hope, and meaning during difficult times. Religious coping can take many forms, including prayer, meditation, attending religious services, reading sacred texts, seeking guidance from religious leaders and fellows, and engaging in religious or spiritual practices such as fasting or pilgrimage (Koenig, 2010; Pargament, 2001; Park, 2005).

Research suggests that religious coping can positively and negatively affect mental health and well-being (Ano & Vasconcelles, 2005; Cheng & Ying, 2023; Pargament et al., 1998; Schwalm et al., 2022). On the one hand, religious coping can provide individuals with a sense of meaning, purpose, and social support, which can promote resilience and help them to cope with stressors. However, on the other hand, some forms of religious coping are associated with negative outcomes, such as increased anxiety, awe, guilt, or feelings of inadequacy.

Our results raise the possibility that individuals with RSP used negative religious coping strategies, whereas the control group, including participants with solid religious beliefs without RSP, were characterized by positive religious coping. The opposite neural and physiological changes in these groups may be related to negative and positive religious coping. However, we did not assess religious coping strategies with separate questionnaires, and therefore, this speculation remains a hypothesis for further studies.

## Limitations

There are several limitations to consider during the interpretation of our results. First, as mentioned above, religious coping strategies were not assessed. Second, the sample was confined to a relatively small number of help-seeking individuals. To improve statistical power and to perform correlational and mediation analyses among behavioral parameters, physiological measures, and brain activity, we need larger representative samples in which scales for religious coping and struggle are administered. Third, we need long-term data on the mental and physical health of the participants, which warrants future studies to focus on the direct relationship between RSP, physiological changes, health status, and well-being. Fourth, to avoid type 2 errors, we strictly reduced the variables according to the main hypotheses. For example, only heart rate indexed cardiovascular reactivity, and we did not measure pro-inflammatory cytokines during the stress response.

## Conclusions

Religious individuals display a marked reduction of stress responses elicited by a social-evaluative situation when reading the Bible and listening to sacred music, as indicated by subjective anxiety reports, physiological changes, and brain activation. However, when RSP is present, religious context does not reduce stress. Instead, it may paradoxically affect physiological responses (heart rate and saliva cortisol) and brain activity (lateralized frontal activation). Therefore, increased physiological stress reactivity in a religious context should be considered during the pastoral care of individuals with RSP.

## References

[CR1] Ai AL, Pargament KI, Appel HB, Kronfol Z (2010). Depression following open-heart surgery: A path model involving interleukin-6, spiritual struggle, and hope under preoperative distress. Journal of Clinical Psychology.

[CR2] Ai AL, Pargament K, Kronfol Z, Tice TN, Appel H (2010). Pathways to postoperative hostility in cardiac patients: Mediation of coping, spiritual struggle and interleukin-6. Journal of Health Psychology.

[CR3] Allen JJ, Cohen MX (2010). Deconstructing the "resting" state: Exploring the temporal dynamics of frontal alpha asymmetry as an endophenotype for depression. Frontiers in Human Neuroscience.

[CR4] American Psychiatric Association (2013). Diagnostic and statistical manual of mental disorders (5th ed.).

[CR5] American Psychiatric Association (2022). Diagnostic and statistical manual of mental disorders (5th ed., text rev.).

[CR6] Ano GG, Pargament KI (2013). Predictors of spiritual struggles: An exploratory study. Mental Health, Religion & Culture.

[CR7] Ano GG, Vasconcelles EB (2005). Religious coping and psychological adjustment to stress: A meta-analysis. Journal of Clinical Psychology.

[CR8] Bali A, Jaggi AS (2015). Clinical experimental stress studies: Methods and assessment. Reviews in the Neurosciences.

[CR9] Beck AT, Epstein N, Brown G, Steer RA (1988). An inventory for measuring clinical anxiety: Psychometric properties. Journal of Consultant and Clinical Psychology.

[CR10] Beck AT, Steer RA, Ball R, Ranieri W (1996). Comparison of beck depression inventories - IA and -II in psychiatric outpatients. Journal of Personality Assessment.

[CR11] Carter WR, Johnson MC, Borkovec TD (1986). Worry: an electrocortical analysis. Advances in Behaviour Research and Therapy.

[CR12] Cheng C, Ying W (2023). A meta-analytic review of the associations between dimensions of religious coping and psychological symptoms during the first wave of the COVID-19 pandemic. Frontiers in Psychiatry.

[CR13] Coan JA, Allen JJ (2004). Frontal EEG asymmetry as a moderator and mediator of emotion. Biological Psychology.

[CR14] Davidson RJ (2004). What does the prefrontal cortex "do" in affect: Perspectives on frontal EEG asymmetry research. Biological Psychology.

[CR15] Dickerson SS, Kemeny ME (2004). Acute stressors and cortisol responses: A theoretical integration and synthesis of laboratory research. Psychological Bulletin.

[CR16] Duan H, Fang H, Zhang Y, Shi X, Zhang L (2019). Associations between cortisol awakening response and resting electroencephalograph asymmetry. PeerJ.

[CR17] Düsing R, Tops M, Radtke EL, Kuhl J, Quirin M (2016). Relative frontal brain asymmetry and cortisol release after social stress: The role of action orientation. Biological Psychology.

[CR18] Engels AS, Heller W, Mohanty A, Herrington JD, Banich MT, Webb AG, Miller GA (2007). Specificity of regional brain activity in anxiety types during emotion processing. Psychophysiology.

[CR19] Exline JJ, Pargament KI, Grubbs JB, Yali AM (2014). The Religious and Spiritual Struggles Scale: Development and initial validation. Psychology of Religion and Spirituality.

[CR20] Exline JJ, Rose E, Paloutzian RF, Park CL (2005). Religious and spiritual struggles. Handbook of the Psychology of Religion and Spirituality.

[CR21] First MB, Williams JBW, Karg RS, Spitzer RL (2016). Structured Clinical Interview for DSM-5 Disorders—Clinician Version (SCID-5-CV).

[CR22] Greenfield EA, Marks NF (2007). Religious social identity as an explanatory factor for associations between more frequent formal religious participation and psychological well-being. International Journal for the Psychology of Religion.

[CR23] Grof, C., & Grof, S. (1990). *The Stormy Search for the Self: a Guide to Personal Growth through Transformational Crisis.* J.P. Tarcher.

[CR24] Haehl W, Mirifar A, Quirin M, Beckmann J (2021). Differentiating reactivity and regulation: evidence for a role of prefrontal asymmetry in affect regulation. Biological Psychology.

[CR25] Hall EC (1997). Identity religion and values.

[CR26] Hollingshead AA (1975). Four-Factor Index of Social Status.

[CR27] Ironson G, Solomon GF, Balbin EG, O'Cleirigh C, George A, Kumar M, Larson D, Woods TE (2002). The Ironson-Woods Spirituality/Religiousness Index is associated with long survival, health behaviors, less distress, and low cortisol in people with HIV/AIDS. Annals of Behavioral Medicine.

[CR28] Jones AE, Elliott M (2017). Examining social desirability in measures of religion and spirituality using the bogus pipeline. Review of Religious Research.

[CR29] Koenig HG (2010). Spirituality and mental health. International Journal of Applied Psychoanalytic Studies.

[CR30] Koenig HG, Büssing A (2010). The Duke University Religion Index (DUREL): A five-item measure for use in epidemological studies. Religions.

[CR31] Koenig HG, King D, Carson VB (2012). Handbook of religion and health.

[CR32] Lukoff D (1998). From spiritual emergency to spiritual problem: The transpersonal roots of the new DSM-IV category. Journal of Humanistic Psychology.

[CR33] Ma Y, Peng H, Liu H, Gu R, Peng X, Wu J (2021). Alpha frontal asymmetry underlies individual differences in reactivity to acute psychosocial stress in males. Psychophysiology.

[CR34] Magyar-Russell G, Brown IT, Edara IR, Smith MT, Marine JE, Ziegelstein RC (2014). In search of serenity: Religious struggle among patients hospitalized for suspected acute coronary syndrome. Journal of Religion and Health.

[CR35] Musselman, D. (2019). *Hillsong - Two Hours of Worship Piano.* https://www.youtube.com/watch?v=Q04XE2-XhyA.

[CR36] Narvaez Linares NF, Charron V, Ouimet AJ, Labelle PR, Plamondon H (2020). A systematic review of the Trier Social Stress Test methodology: Issues in promoting study comparison and replicable research. Neurobiology of Stress.

[CR37] Pargament, K. I. (2001). *The psychology of religion and coping.* Guilford.

[CR38] Pargament, K. I., & Exline, J. J. (2021). Religious and spiritual struggles and mental health: implications for clinical practice. In *Spirituality and Mental Health Across Cultures.* (pp. 395–412). Oxford University Press. 10.1093/med/9780198846833.003.0024

[CR39] Pargament KI, Exline JJ (2022). Working with spiritual struggles in psychotherapy: From research to practice.

[CR40] Pargament K, Feuille M, Burdzy D (2011). The Brief RCOPE: current psychometric status of a short measure of religious coping. Religions.

[CR41] Pargament KI, Koenig HG, Tarakeshwar N, Hahn J (2001). Religious struggle as a predictor of mortality among medically ill elderly patients: A 2-year longitudinal study. Archives of Internal Medicine.

[CR42] Pargament KI, Magyar GM, Benore E, Mahoney A (2005). Sacrilege: A Study of sacred loss and desecration and their implications for health and well-being in a community sample. Journal for the Scientific Study of Religion.

[CR43] Pargament KI, Murray-Swank N, Magyar GM, Ano GG, Miller WR, Delaney H (2004). Spiritual struggle: A phenomenon of interest to psychology and religion. Judeo-Christian Perspectives in Psychology: Human Nature, Motivation, and Change.

[CR44] Pargament KI, Smith BW, Koenig HG, Perez L (1998). Patterns of positive and negative religious coping with major life stressors. Journal for the Scientific Study of Religion.

[CR45] Park CL (2005). Religion as a meaning-making framework in coping with life stress. Journal of Social Issues.

[CR46] Perczel-Forintos, D., Ajtay, G., Barna, C., Kiss, Z., & Komlósi, S. (2018). *Kérdőívek, becslőskálás a klinikai pszichológiában.* Medicina.

[CR47] Pitchford B, Arnell KM (2019). Self-control and its influence on global/local processing: An investigation of the role of frontal alpha asymmetry and dispositional approach tendencies. Attention, Perception, & Psychophysics.

[CR48] Pomerleau JM, Pargament KI, Krause N, Ironson G, Hill P (2020). Religious and spiritual struggles as a mediator of the link between stressful life events and psychological adjustment in a nationwide sample. Psychology of Religion and Spirituality.

[CR49] Prusak J (2016). Differential diagnosis of Religious or Spiritual Problem possibilities and limitations implied by the V-code 6289 in DSM-5. Psychiatria Polska.

[CR50] Ramirez SP, Macêdo DS, Sales PM, Figueiredo SM, Daher EF, Araújo SM, Pargament KI, Hyphantis TN, Carvalho AF (2012). The relationship between religious coping, psychological distress and quality of life in hemodialysis patients. Journal of Psychosomatic Research.

[CR51] Reznik SJ, Allen JJB (2018). Frontal asymmetry as a mediator and moderator of emotion: an updated review. Psychophysiology.

[CR52] Roth S, Cohen LJ (1986). Approach, avoidance, and coping with stress. American Psychologist.

[CR53] Rózsa S, Kő N, Mészáros A, Kuncz E, Mlinkó R (2010). Wechsler Felnőtt Intelligenciateszt - negyedik kiadás.

[CR54] Sapolsky RM (2021). Glucocorticoids, the evolution of the stress-response, and the primate predicament. Neurobiology of Stress.

[CR55] Schnell T, Fuchs D, Hefti R (2020). Worldview under stress: Preliminary findings on cardiovascular and cortisol stress responses predicted by secularity, religiosity, spirituality, and existential search. Journal of Religion and Health.

[CR56] Schwalm FD, Zandavalli RB, de Castro Filho ED, Lucchetti G (2022). Is there a relationship between spirituality/religiosity and resilience? A systematic review and meta-analysis of observational studies. Journal of Health Psychology.

[CR57] Sephton SE, Koopman C, Schaal M, Thoresen C, Spiegel D (2001). Spiritual expression and immune status in women with metastatic breast cancer: An exploratory study. Breast Journal.

[CR58] Stauner N, Exline JJ, Grubbs JB, Pargament KI, Bradley DF, Uzdavines A (2016). Bifactor models of religious and spiritual struggles: distinct from religiousness and distress. Religions.

[CR59] Tobin ET, Slatcher RB (2016). Religious participation predicts diurnal cortisol profiles 10 years later via lower levels of religious struggle. Health Psychology.

[CR60] Tops M, Quirin M, Boksem MAS, Koole SL (2017). Large-scale neural networks and the lateralization of motivation and emotion. International Journal of Psychophysiology.

[CR61] Urry HL, Nitschke JB, Dolski I, Jackson DC, Dalton KM, Mueller CJ, Rosenkranz MA, Ryff CD, Singer BH, Davidson RJ (2004). Making a life worth living: Neural correlates of well-being. Psychological Science.

[CR62] Wechsler D (2008). Wechsler adult intelligence scale.

[CR63] Zhang X, Bachmann P, Schilling TM, Naumann E, Schachinger H, Larra MF (2018). Emotional stress regulation: The role of relative frontal alpha asymmetry in shaping the stress response. Biological Psychology.

[CR64] Zondervan. (2019). The God of comfort: 100 Bible verses to soothe our spirit*.* Zondervan

